# BIOPSYCHOSOCIAL NATURAL HISTORY OF ALS: INTRODUCTION TO THE OPEN DATA, ACCESS, AND USE

**DOI:** 10.1093/geroni/igae098.2689

**Published:** 2024-12-31

**Authors:** Suzanne Segerstrom, Edward Kasarskis

**Affiliations:** Oregon State University, Corvallis, Oregon, United States; University of Kentucky, Lexington, Kentucky, United States

## Abstract

The Seattle Amyotrophic Lateral Sclerosis (ALS) Patient Profile Database is a rich longitudinal dataset of ALS patients (n = 143; ages 26-83 years) and their partners (spouses, significant others, or caregivers; n = 123; ages 20-79 years) from clinics in Seattle, WA; San Francisco, CA; and Philadelphia, PA. The purpose of the study was to characterize the psychosocial and physical natural history of ALS and the interactions between them. Participants and partners (84% spouses) were separately interviewed in their homes every 3 months for up to 18 months between March 1987 and August 1989, with survival data collected up to 2008. Psychological measures include social cognition (e.g., self-perception and informant perception), psychological processes (e.g., coping, emotional expression), psychological health (e.g., anger, depression), social role activities (e.g., work, church attendance), and social support. Disease history, severity, progression, and survival data are available along with health behavior history, use of respirator and feeding tube, treatments (including psychotherapy or counseling), and medical and family history. This database is newly available and open on Mendeley Data, providing the opportunity for scientists to test biopsychosocial hypotheses in this hard-to-obtain patient population. Much of the data has never been analyzed or reported. In this presentation, the structure and contents of the dataset, the means of access, and the handful of previous findings will be introduced to the GSA meeting attendees.

